# Review Article: Tools and trends for probing brain neurochemistry

**DOI:** 10.1116/1.5051047

**Published:** 2019-06-11

**Authors:** Abraham G. Beyene, Sarah J. Yang, Markita P. Landry

**Affiliations:** 1 Department of Chemical and Biomolecular Engineering, University of California, Berkeley, Berkeley, California 94720; 2 Helen Wills Neuroscience Institute, University of California, Berkeley, Berkeley, California 94720; 3 California Institute for Quantitative Biosciences (qb3), Berkeley, California 94720; 4 Chan-Zuckerberg Biohub, San Francisco, California 94158

## Abstract

The brain is composed of complex neuronal networks that interact on spatial and temporal scales that span several orders of magnitude. Uncovering how this circuitry gives rise to multifaceted phenomena such as perception, memory, and behavior remains one of the grand challenges in science today. A wide range of investigative methods have been developed to delve deeper into the inner workings of the brain, spanning the realms of molecular biology, genetics, chemistry, optics, and engineering, thereby forming a nexus of discovery that has accelerated our understanding of the brain. Whereas neuronal electrical excitability is a hallmark property of neurons, chemical signaling between neurons—mediated by hundreds of neurotransmitters, neuromodulators, hormones, and other signaling molecules—is equally important, but far more elusive in its regulation of brain function for motor control, learning, and behavior. To date, the brain's neurochemical state has been interrogated using classical tools borrowed from analytical chemistry, such as liquid chromatography and amperometry, and more recently, newly developed fluorescent sensors. Here, the authors review advances in the development of functional fluorescent probes that are beginning to expand their understanding of the neurochemical basis of brain function alongside device-based analytical tools that have already made extensive contributions to the field. The emphasis herein is on the paradigms of probe and device development, which follow certain design principles unique to the interrogation of brain chemistry.

## I. INTRODUCTION

Neurons are the fundamental units of the nervous system. Thus, understanding neural activity at commensurate spatial and temporal scales is paramount to furthering fundamental neuroscience research. The adult human brain is estimated to contain in excess of 80 × 10^9^ neurons making several trillion synaptic connections. The interactions that emerge from this vast and complex network of neurons give rise to complex behavioral outcomes such as perception, memory, and cognition. Gaining fundamental insight into how neuronal networks give rise to emergent phenomena remains a grand challenge in neuroscience.

Classically, the study of the brain has been approached from one of three perspectives: (i) the study of neuron structure and connectivity, (ii) the study of neuronal electrical activity, and (iii) the study of chemical neurotransmission between neurons. One of the earliest explorations in neuroanatomy utilized Golgi's silver staining method to visualize neuronal connectivity and eventually gave rise to the neuronal doctrine of modern neuroscience. Advances in subsequent decades were led by developments in electrophysiology, which gave rise to a host of electrode-based techniques, such as whole cell patch clamp and amperometry, which have enabled the investigation of the electrophysiological properties of neurons. While these methods are among the most established within neuroscience, they are limited in their capacity to interrogate large-scale neuronal activity. More recently, fluorescence microscopy has enabled interrogation of the structural connectivity, electrical activity, and chemical neurotransmission between systems of neurons. This is enabled through the creation of probes that access the relevant spatial and temporal scales of each perspective. Herein, we review recent developments in probes to study brain function with particular focus on the nascent field of developing fluorescent probes to query brain chemical neurotransmission.

### A. Fluorescence microscopy in neuroscience research

Fluorescent probes can be designed to either directly or indirectly report on the structure, electrical activity, or chemical activity of neurons. Typically used in conjunction with microscopes, these probes are capable of imaging not only the underlying cellular structures and connectivity of neurons but also the resident immune cells of the brain including microglia and astrocytes. Thus, advances in optical microscopy and computation, advances in genetics, and growing libraries of fluorescent markers have contributed to detailed mapping of cellular and subcellular organization of neurons and their dynamic morphological changes over multiple spatial and temporal scales. As a result, fluorescent strategies toward elucidating neuron structure and organization have emerged as an established cornerstone of modern *in vitro* and *in vivo* neuroscience research.

In contrast to probes developed to study brain structure, functional probes that explore neuronal electrical and chemical activity often must satisfy a different set of design principles and are coupled with advanced light microscopy technologies. Fluorescent molecules employed for structural imaging serve as contrast agents, enabling diffraction-limited imaging of structural components that display the fluorescent molecules. Conversely, functional probes can be engineered to undergo modulations in fluorescence in response to selective biochemical events in their vicinity, typically in the form of changes in fluorescence quantum yield (hence fluorescence intensity), radiative decay lifetimes, fluorescence energy transfer, or fluorescence quenching of the probe. These modulations can be imaged over time, producing a video-rate sequence of images that recapitulate the underlying transients in the local neurochemistry. The most prominent and widely used functional fluorescent probes in basic neuroscience research either respond to calcium ions (Ca^2+^) or membrane voltage changes (voltage-sensitive). These probes are typically used to study action potentials, the logic-gated electrical signals used by neurons to transmit information.

Calcium probes provide indirect information about action potentials by undergoing fluorescence modulation upon binding to calcium ions. As such, calcium probes provide a real-time readout of Ca^2+^ concentration fluctuations arising from intermittent opening and closing of voltage or ligand-gated calcium channels. These concentrations serve as a proxy of actual electrical activity. Conversely, voltage-sensitive probes undergo fluorescence modulation in direct response to membrane voltage changes by localizing on the neuronal plasma membrane and responding fluorescently to voltage transients. This allows voltage-sensitive probes to effectively capture action potential temporal dynamics. However, despite their widespread use, calcium or voltage sensors alone do not provide a complete picture of brain activity. As we discuss below, chemical signaling between neurons, despite being a direct consequence of electrophysiological activity, is a distinct phenomenon that requires a separate class of functional fluorescent probes for imaging.

### B. Basics of chemical neurotransmission

**Fig. 1.**
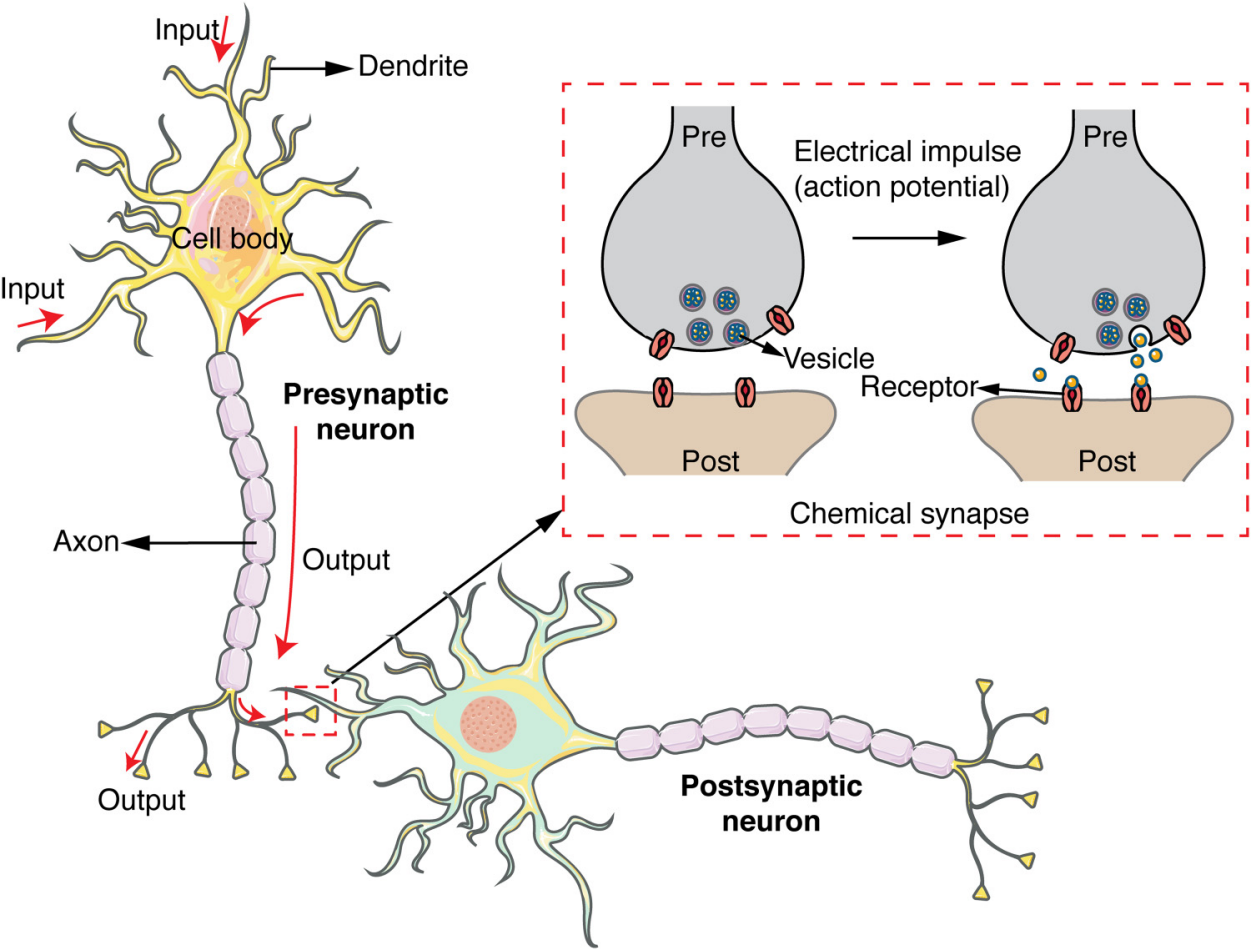
Chemical signaling between a presynaptic and postsynaptic neuron. An action potential carries electrical signal down to axon terminals. Chemical synapses form between the presynaptic and postsynaptic neuron convert the action potential into a chemical signal that can travel across the synaptic cleft.

While action potentials rapidly transfer information down a nerve fiber within a single neuron, communication between neurons requires the release of chemical signaling molecules across the synaptic cleft that separates the two cells (Fig. 1). Upon reaching the synaptic terminal, action potentials trigger a series of complex biochemical steps in the presynaptic neuron that culminate in the exocytotic release of chemical signaling molecules into the synaptic cleft. Once released, classical neurotransmitters such as glutamate and γ-aminobutyric acid (GABA) travel across the synaptic cleft and bind to ligand-gated ion channels located on the postsynaptic cell. This binding induces rapid excitatory or inhibitory currents in the postsynaptic cell that completes the communication between the presynaptic and postsynaptic neurons and may further facilitate or inhibit the generation of a new action potential. Neurotransmitters can also operate by modulating the electrophysiological properties of their target neurons rather than inducing a direct electrical current. These neurotransmitters, known as neuromodulators, bind to G-protein coupled receptors (GPCRs) and act indirectly—and relatively slowly—by modulating the intrinsic excitability of their target neurons. Regardless of neurotransmitter type, the release of neurotransmitters in response to electrical activity is a highly stochastic process that does not exhibit an exact correspondence with electrophysiological activity. Therefore, even if the electrophysiological signaling of complex neuronal networks can be characterized, the underlying chemical neurotransmission is distinct and equally daunting in its complexity. Accordingly, there is a need for a unique set of fluorescent sensors that provide chemical signaling readout and directly query chemical neurotransmission. We seek to highlight the tools that have been developed in response to this need and opened up a new dimension of information around neuronal activity and wiring, and ultimately, brain function.

### C. Imaging neuromodulation: Challenge of chemical selectivity

Although calcium and voltage transients are conserved entities, neurons communicate by releasing a large array of chemical signaling molecules, including neurotransmitters, neuromodulators, neuropeptides, and hormones. To date, roughly 100 different chemical signaling molecules have been identified. The most common molecules, referred to as neurotransmitters, include glutamate and GABA, respectively, as the brain's primary excitatory and inhibitory molecules. Molecules such as dopamine (DA), serotonin, and a wide array of neuropeptides are collectively referred to as neuromodulators. These molecules “tune” the electrophysiological properties of their target neurons by making them more or less excitable and “modulate” the effect that the excitatory glutamate and the inhibitory GABA exert on their targets. The rich diversity of chemical signaling molecules and their signaling pathways add an additional challenge to developing selective probes.

The ideal fluorescent probe would exhibit a selective affinity for its target over competing neurotransmitters, precursors, and metabolites; optimal binding kinetics to enable good temporal resolution; high spatial resolution of recording; resistance to biofouling; and biostability for long-term use. Furthermore, the probe's dissociation constant (K_d_) needs to be optimized for imaging in particular brain subregions in order to accommodate the heterogeneity in concentrations and release/reuptake profiles, and contrary to intuition, high binding affinities are not always desirable for optimal temporal resolution.^1^ However, simultaneously satisfying every element in this list of criteria is a daunting task. Nevertheless, developments in protein engineering, precise genetic manipulation, synthetic small fluorescent molecules, and nanoparticle synthesis have enabled nascent development of novel probes that can assay neurochemistry with spatial and temporal resolutions that match the requisite scales for neuroscience research. Here, we provide a review of such sensors and probes that have been developed for measuring the spatiotemporal dynamics of chemical signaling molecules in the brain, with a particular emphasis on the approaches employed for sensor development. Within this larger direction, we first discuss protein-based probes that are based on fluorescent proteins conjugated to molecular recognition domains, followed by a discussion on synthetic fluorescent probes that employ synthetic fluorescent molecules and nanoparticles. Finally, we discuss device-based approaches such as microdialysis that adapt established benchtop analytical techniques for the purposes of probing neurochemistry.

## II. PROTEIN-BASED FLUORESCENT PROBES

Protein-based fluorescent probes are characterized by their use of protein constructs to bind neurotransmitters and generate an optical signal. This is often achieved by mutating existing proteins with natural binding affinity for the desired neurochemical analyte to design fluorescent probes that generate precise spatial and temporal information. In a typical protein-based fluorescent probe, the protein-based analyte recognition moiety is covalently conjugated to a fluorophore. During analyte binding, the recognition domain undergoes a conformation change that perturbs the photophysical properties of the conjoined fluorophore, providing a readout for the recognition event. The fluorophore for a protein-based neurotransmitter sensor is not necessarily a fluorescent protein, as there are a number that employ synthetic fluorophores. However, the use of fluorescent proteins is convenient in that no additional synthetic materials need be introduced after transgenic expression of the fluorescent protein.^2^

A major advantage of using protein-based neurotransmitter sensors is the ability to create genetically encoded sensors that target specific cell populations or compartments.^3^ Delivery of transgenic DNA encoding the protein-based sensor is readily accomplished using established and relatively noninvasive delivery mechanisms such as transgenesis or viral vectors.^3^ In a similar vein, protein-based neurotransmitter sensor performance is often characterized *in vitro* in cell cultures, *ex vivo* in neuronal tissue, or even *in vivo* in behaving animals. This ensures that sensor performance is maintained even in the presence of a complex biological milieu and that the sensor itself is compatible with a living, biological system. We further sort protein-based fluorescent probes into two categories based on their signal generation strategies: Forster resonance energy transfer (FRET)-based protein sensors and single wavelength protein sensors. Each strategy holds its own advantages and drawbacks, further delineated below.

### A. FRET-based protein sensors

Reporting on biochemical events via changes in FRET is a widely established starting principle for sensor design.^2^ FRET is a form of nonradiative energy transfer that can occur between two fluorophores in close proximity with overlapping absorption and emission spectra. The FRET efficiency between a pair of fluorophores (referred to as donor and acceptor fluorophores) is ultrasensitive to the distance between them, affording a precise readout of nanometer-scale conformational changes that follow protein-ligand binding events. Though many variations exist, FRET-based probes typically seek to leverage biochemically driven changes in spatial conformation to induce changes in FRET efficiency between donor and acceptor fluorophores. A major appeal of FRET-based sensors is their ratiometric nature, which provides quantitative information on the kinetics of observed process that can be gleaned from the fluorescent output.^4^ However, this necessitates sensors that are highly sensitive to changing environmental cues. Furthermore, the dual-fluorophore design that enables ratiometric reporting from FRET-based sensors requires larger spectral bandwidth than a single wavelength method. This results in challenges when attempting to multiplex FRET sensors, particularly protein-based FRET sensors, which often draw from fluorophores with contiguous fluorescent properties.^5^

#### 1. Fluorescent indicator protein for glutamate

**Fig. 2.**
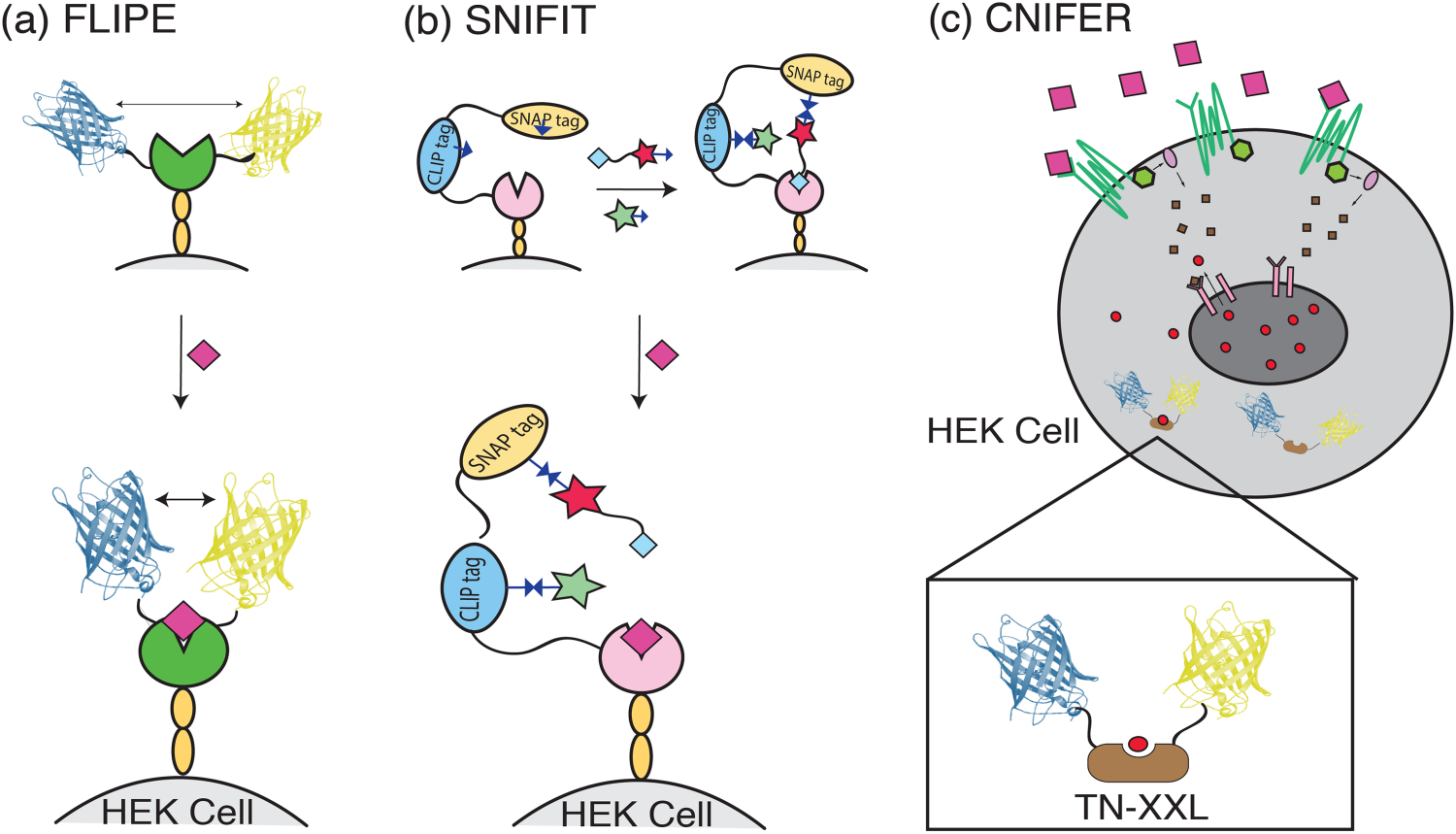
Schematic of FRET-based protein sensors mechanisms. (a) FLIPE expressed on the cell surface undergoes a “hinge-bending” motion in YbeJ (opened circle) in the presence of glutamate (diamond) to bring ECFP and VENUS closer together (b) SNIFIT protein construct expressed on the cell surface is “clicked” to DY-547 (star) and Cy5 (star tethered to glutamate analog). Without glutamate, the glutamate analog bound to Cy5 subsequently binds the S1S2 domain of the iGluR5 receptor. Addition of glutamate (diamond) outcompetes the glutamate analog and allows the dyes to separate, thereby changing the FRET efficiency. (c) CNiFER cells expressing the relevant receptor bind extracellular dopamine (large squares outside the cell) and initiate intracellular calcium release (small circles inside the cell) through a signaling cascade (small squares inside the cell). FRET sensor TN-XXL binds the released calcium and generates an FRET signal.

One of the earliest generations of an FRET-based protein sensor was the fluorescent indicator protein for glutamate (FLIPE) developed by Okumoto *et al.* by fusing enhanced cyan fluorescent protein (ECFP) and VENUS, a yellow fluorescent protein, to the opposite ends of the glutamate-binding bacteria periplasmic protein YbeJ.^6^ The presence and subsequent binding of glutamate initiate a “hinge-bending” motion in YbeJ that brings ECFP and VENUS together and generates a change in FRET efficiency [Fig. 2(a)]. Multiple FLIPE variants have been developed across a range of binding affinities, most notably FLIPE-600n and FLIPE-10μ which have a dissociation constant (K_d_) of 600 nM and 10 *μ*M, respectively. To localize FLIPE at the cell surface, the FRET-YbeJ construct is inserted into a pDisplay plasmid, which expresses the sensor protein between two sequences that anchor and display the sensor to the extracellular plasma membrane of the cell of interest. When expressed on the membrane of PC12 cells, a model progenitor neuronal cell line, FLIPE-600n demonstrates a dynamic range between 100 nM and 1 *μ*M and generates a moderate maximum ratio change (ΔRmax) of 0.27 *in vitro.*^6^ This moderate fluorescence response is a challenge when employing FLIPE to study neurotransmitter signaling events, since probe signal and imaging frame rate (i.e., temporal resolution) are inversely related. Kinetic studies based on *E. coli* glutamine and histadine binding proteins suggest that YbeJ should exhibit binding and dissociation kinetics on the order of ∼1 ms.^7^ This temporal resolution is within the temporal resolution necessary for studying synaptic release phenomena. However, no extensive kinetic studies on the bound FLIPE construct have been conducted to measure synaptic neurotransmission with the current FLIPE system. In addition, initial studies spanning a time of ∼30 min have observed a decrease of VENUS emission over the course of the experiment, which could limit the experimental time-window over which such probes can be employed.^6^

#### 2. Semisynthetic fluorescent sensor protein

The semisynthetic fluorescent sensor protein (Snifit) system was developed in response to the moderate glutamate sensitivity demonstrated by FLIPE. Snifit employs the S1S2 glutamate-binding domain of the ionotropic glutamate receptor 5 (iGluR5) as a glutamate recognition element and expresses this domain in linear fusion with an SNAP tag and CLIP tag, which serve as fusion protein sites that allow for orthogonal covalent attachment of fluorophores. The combined fusion protein can then be displayed on the surface membrane of HEK 293T cells via the pDisplay system. In contrast to FLIPE, Snifit utilizes the artificial fluorescent dyes DY-547 and Cy5, covalently attached to the CLIP tag and SNAP tag, respectively, in place of fluorescent proteins to increase the *ex vivo* ΔRmax to 1.56 [Fig. 2(b)].^8^ This ΔRmax is achieved by attaching the artificial dyes using straightforward click chemistry associated with the SNAP and CLIP tag systems following fusion protein expression on the cell surface. Further increases in ΔRmax for the SNIFIT system can be achieved by replacing the CLIP tag with a fluorescent unnatural amino acid (uSNIFIT).^9^ This ability to maintain the cell specificity of a genetically encoded sensor while also leveraging nonfluorescent protein fluorophores to increase probe sensitivity is a major appeal of the SNIFIT system. In addition, the SNIFIT system has been developed for a wide range of targets, including the neurotransmitters GABA and acetylcholine (Ach).^10,11^ Though the current literature only demonstrates the neurotransmitter sensing abilities of SNIFIT for *in vitro* applications in cell lines, a similar protein-SNAP system has been demonstrated in live brain tissue, where tissue from mice expressing the protein-SNAP fusion is subsequently labeled with fluorophore *ex vivo* and imaged.^12^ Kinetic studies of natural iGluR5 glutamate interaction suggest binding and dissociation kinetics on the order of ∼1 ms.^8^ However, these kinetics have not been verified for the membrane-bound SNIFIT construct.

#### 3. Cell-based neurotransmitter fluorescent engineered reporters

Despite the gains in probe sensitivity made by the SNIFIT system, a number of challenges remain in FRET-based protein sensors, namely, low analyte sensitivity in the nanomolar range, difficulty in developing sensors for neurotransmitters such as dopamine and norepinephrine (NE) that signal through GPCRs, and lack of robust experiments *in vivo.*^13^ Cell-based neurotransmitter fluorescent engineered reporters (CNiFERs) seek to meet these challenges by adopting a different approach from the SNIFIT and FLIPE sensors. Rather than building a sensor around a conformation-changing-protein, CNiFERs are clonal HEK293 cells specially engineered to express a natural GPCR that increases intracellular Ca^2+^ concentration upon binding its endogenous ligand [Fig. 2(c)]. This increase in intracellular Ca^2+^ is subsequently detected by a genetically encoded FRET Ca^2+^ sensor TN-XXL which operates via cyan and yellow fluorescent protein fluorophores [Fig. 2(c)].^13^

To date, CNiFER systems have been developed for sensing Ach via the M1 muscarinic receptor, DA via the D2 receptor, and NE via the *α*_1A_ receptor. CNiFER response tests *in vitro* indicate that the sensor provides a phasic response within ∼20–40 s of bolus neurotransmitter delivery, followed by a tonic plateau that stabilizes after ∼300 s.^14^ The phasic response has been shown to be independent of external calcium concentration, while the tonic response is eliminated in calcium-free media.^14^ The effective concentration for 50% response level (EC_50_) of the phasic response is reported at 11 nM for M1-CNiFER, 2.5 nM for D2-CNiFER, and 19 nM for *α*_1A_-CNiFER. This indicates optimal sensor sensitivity within the natural concentration range of each neurotransmitter measured via existing methods such as microdialysis and FSCV.^14,15^ Of particular note is the difference in dopamine and norepinephrine sensitivity between D2-CNiFER and *α*_1A_-CNiFER. Historically, dopamine and norepinephrine signals have been difficult to distinguish from one another due to their structural similarity.^16^ The CNiFER system overcomes this challenge by leveraging the natural selectivity of the D2 and *α*_1A_ GPCRs for dopamine and norepinephrine, respectively. As a result, D2-CNiFER shows an ∼30-fold drop in sensitivity for norepinephrine compared to dopamine, and *α*_1A_-CNiFER exhibits sensitivity for dopamine only at high dopamine concentrations approaching the micromolar range.

CNiFER system's use of natural receptors allows it to leverage the natural binding and dissociation kinetics of neurotransmitter receptors. However, its temporal resolution is reliant on the activation of the inositol triphosphate (IP_3_) signaling pathway, resulting in an ∼2 s delay in the signal after stimulus delivery.^14,15^ Experiments that expose CNiFER cells to pulses of neurotransmitter *in vitro* show that M1-CNiFER responds to 2.5 s pulses of 100 nM Ach with a relative fluorescence of ΔF_max_/F** **=** **0.9 and is capable of separating pulses with an interstimulus of 6 s.^14^ Similarly, D2-CNiFER responds to 2.5 s pulses of 100 nM DA with a maximum FRET ratio ΔR/R** **=** **0.57, and *α*_1A_-CNiFER responds to 2.5 s pulses of 100 nM NE with a maximum FRET ratio ΔR/R** **=** **0.90. Both D2-CNiFER and *α*_1A_-CNiFER are capable of separating pulses as close as 3 s.^14,15^

Unlike the FLIPE and SNIFIT systems, the CNiFER system has been characterized extensively *in vivo*. After stereotaxic injection into the mouse cortex, implanted CNiFER cells maintain their temporal resolution and neurotransmitter sensitivity and respond to electrically or pharmacologically induced changes neurotransmitter concentrations in the brains of awake and behaving mice.^14,15^ Furthermore, chronically implanted CNiFER cells can be imaged for at least six days postimplantation.^15^ Implantation of two sets of CNiFER cells in spatially distinct regions also allows signals from two different neurotransmitters to be tracked at once.^15^

Despite many strides taken by the CNiFER systems, areas of improvement remain. When developing new CNiFER cells for new neurotransmitters, finding receptor variants with adequate sensitivity and resistance to desensitization is of paramount importance.^13^ This is partially ameliorated by the modular design of the CNiFER system which allows for different Ca^2+^ sensors or GPCR receptors to be readily substituted in.^13^ More profoundly, the CNiFER system was developed with a desire to specifically examine volume transmission in the brain. By this metric, the CNiFER system's spatial resolution is suboptimal and cannot resolve single synapse neurotransmission events. Similarly, CNiFER's temporal resolution is limited by its dependence on the generation of Ca^2+^ concentration changes via indirect cellular signaling pathways. Thus, the instantaneous sensor response remains to be achieved. Nevertheless, CNiFER cells remain among the most robustly characterized methods for monitoring of neurotransmitters—particularly neuromodulators—*in vivo*.

### B. Single wavelength fluorescent reporters

In contrast to FRET-based sensors, single wavelength sensors relay an optical signal via changes in a single fluorophore, rather than changes in donor-acceptor pair FRET efficiency. This readout results in a sensor with relatively compact optical excitation and emission spectra that make them more amenable to multiplexing. Another major difference between FRET and single wavelength sensors is ratiometry, which is often used in FRET systems to determine analyte concentrations. While single wavelength sensors are not ratiometric, they often have greater sensitivity, which can be difficult to achieve in FRET systems where changes in optical signals tend to be low.^3^

#### 1. Glutamate optical sensors

**Fig. 3.**
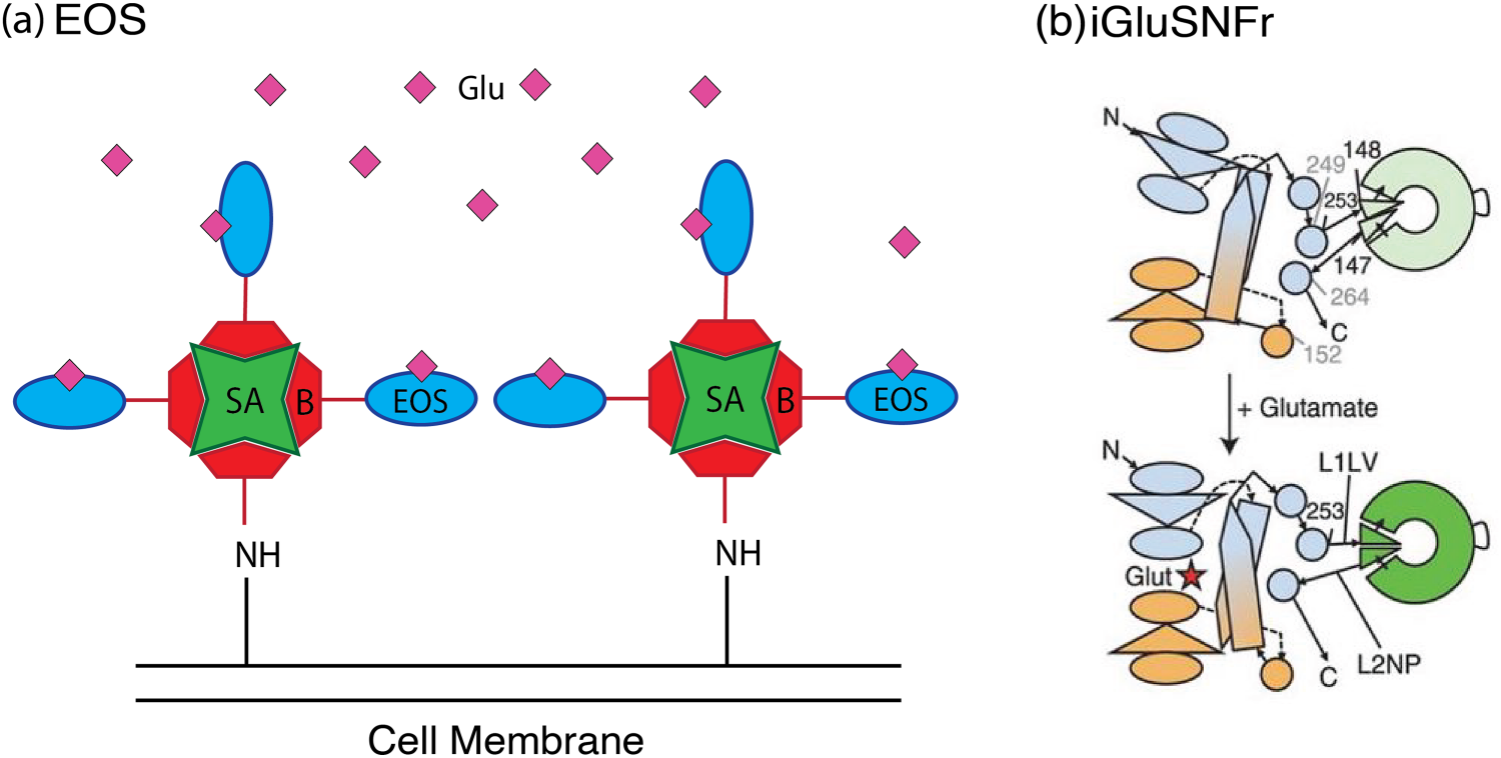
Schematic of single wavelength protein sensor mechanisms. (a) EOS protein constructs are bound to biotin (B) and incubated with streptavidin (SA) coated cells to label the cell surface with sensor. Adapted with permission from S. Namiki, H. Sakamoto, S. Iinuma, M. Iino, and K. Hirose, Eur. J. Neurosci. **25**, 2249 (2007). Copyright 2007, Wiley. (b) iGluSnFR constructs made up of cGFP (disk) inserted into *GltI* typically have disrupted cGFP structures with dim fluorescence. The conformation change in *GltI* caused by glutamate binding structurally realigns cGFP to elicit brighter fluorescence. Reprinted with permission from J. S. Marvin *et al.*, Nat. Methods **10**, 162 (2013). Copyright 2013, Nature Publishing.

The development of the glutamate (amino acid abbreviation E) optical sensor (EOS) sought to improve upon the signal strength of the FRET-based glutamate sensor FLIPE and thus generate a sensor with greater spatial resolution.^17^ EOS is developed from the mutated glutamate receptor GluR2 subunit of the a-amino-3-hydroxy-5-methyl-4-isoxazolepropionic acid (AMPA) receptor. Substituting a cysteine residue at position 403 in the AMPA receptor binding pocket allows for the introduction of the thiol-reactive dye, Oregon green, after the protein is expressed in *E. coli*. The modified protein is then subsequently biotinylated and attached to the surface of similarly biotinylated cells via incubation with streptavidin [Fig. 3(a)]. Cell surface immobilized EOS exhibits a strong change in fluorescence upon glutamate addition, with the most optimized mutants binding glutamate with a dissociation constant (K_d_) of 174 nM and generating a maximal change in fluorescence intensity (ΔF_max_/F) of 29.1% when localized on the cell membrane.^18^ Lower affinity variants for sensing in the micromolar range have also been developed, with K_d_** **=** **1.57 *μ*M and an even larger ΔF_max_/F =** **48.2%.^18^ Kinetically, EOS is characterized by a rapid association rate constant of roughly 107 M^−1^ s^−1^ and a slower apparent dissociation rate constant of 0.67 s^−1^. These dynamics have been argued to be advantageous for imaging, allowing rapid binding of short-lived glutamate release events and “holding” of the signal to enable detection by the imaging apparatus.^17^

EOS glutamate sensing has been characterized both *ex vivo* in brain slices and *in vivo* in awake and behaving mice. In both cases, tissue is labeled with the modified EOS protein using biotin-streptavidin conjugation and good labeling of the brain extracellular matrix is achieved. As a result, the improved spatial resolution of EOS combined with its relatively sharp temporal resolution has allowed interrogation of the effects of glutamate spillover on synaptic activity in mice.

#### 2. Intensity-based glutamate sensing fluorescent reporter

A major difference between the EOS system and the other protein-based sensing methods highlighted in this section is that EOS is not a genetically encoded system. Despite aforementioned strengths, the EOS system does not leverage many of the unique benefits of genetically encoded sensors such as easy targeting of specific cell populations, access to noninvasive sensor delivery techniques, and capacity for repeated imaging over months.^3^ Thus, the intensity-based glutamate sensing fluorescent reporter (iGluSnFR) was developed as a genetically encoded, single wavelength alternative to EOS, as well as to the previously developed FRET-based sensor superGluSnFR. iGluSnFR is composed of a circularly permuted green fluorescent protein (cGFP) inserted into the bacterial periplasmic binding protein, *GltI*, from *E. coli* such that the cGFP structure is disrupted and produces dim fluorescence. Binding of glutamate rectifies this disruption, resulting in a bright GFP signal [Fig. 3(b)].^3^ The DNA for this construct is then incorporated into the pDisplay system to localize the expressed protein onto the cell membrane, as in the FLIPE and SNIFIT systems, and the DNA is delivered to the target cells using an Adeno Associated Viral (AAV) vector. When expressed *in vitro* on the cell surface, iGluSnFR has a K_d_** **=** **4.9 *μ*M and a ΔF_max_/F_0_** **=** **1.03** **±** **0.15 (or ∼103%).^3^ This translates into high spatial resolution during *in vivo* imaging, enabling resolution of individual dendrites in *C. elegans*, larval zebrafish, and mice. In addition, iGluSnFR responds with a precise temporal resolution as demonstrated by ∼5 ms response times during glutamate uncaging experiments.^3^

More recent variants of iGluSnFR have also been developed by substituting the eGFP construct with a circularly permuted “superfolder” GFP (SF-GFP) and mutating select residues at the hinge of *GltI*. This has resulted in new “super folder” iGluSnFR (SF-iGluSnFR) constructs with improved *in vivo* performance due to their brighter signal and higher expression on the cell surface.^19^ Tighter binding SF-iGluSnFR variants with dissociation constants as low as 0.7 *μ*M have also been developed which are capable of resolving glutamate release at individual dendritic spines.^19^ Additionally, in neuronal culture, nonmembrane bound variants with weaker affinity, but faster on and off rate kinetics, have been shown to discern synaptic events with higher temporal resolution than iGluSnFR and GCaMP6f.^19^

#### 3. G-protein coupled-receptor based probes (dLight, GRAB_DA_, GAch)

Given the advantages conferred by the design of iGluSnFR, a similarly directed class of sensors has been developed by inserting circularly permutated GFP into the sequences of endogenous G-protein coupled receptors for the target analyte. This design allows direct coupling of structural changes incurred by analyte binding to fluorescence modulation of inserted cGFP without the additional need to link the binding protein to the pDisplay system. Utilizing natural receptors for the analyte of interest has allowed this system to be readily developed to target neuromodulators such as DA and Ach.

To date, two dopamine sensors have been developed using this design strategy: dLight and GPCR-activation-based-DA (GRAB_DA_) sensor. Both probes were developed after the optimized insertion of cGPF into endogenous receptors for dopamine. The dLight probe has variants constructed by inserting the cGFP module sequence into the third intracellular loop (IL3) of the human dopamine D1, D2, and D4 receptors. dLight1.1 is obtained from a simple cGFP insertion and dLight1.2 contains an additional Phe129A mutation. The two sensors demonstrate a ΔF/F_max_ of 230** **±** **9% and 340** **±** **20%, respectively. These mutants also demonstrated favorable binding kinetics, with apparent affinities for dopamine of 330** **±** **30 and 770** **±** **10 nM. GRAB_DA_ is constructed from the insertion of cEGFP into the IL3 of the human dopamine D_2_ receptor and also has two variants—GRAB_DA1m_ and GRAB_DA1h_—which both demonstrate an ∼90% ΔF_max_/F_0_ response but have differing affinities of 130 and 10 nM, respectively.

When expressed in HEK293T cells, both dLight and GRAB_DA_ show robust expression at the cell surface and good performance. Furthermore, dLight1 shows ∼70-fold and ∼40-fold decreased affinity for norepinephrine and epinephrine over dopamine, while GRAB_DA_ shows a 10-fold decreased affinity for norepinephrine over dopamine. Given dLight and GRAB_DA_'s use of endogenous receptors, there are concerns that additional expression of dLight and GRAB_DA_ on the cell surface could induce changes to the intracellular state if the sensors are linked to the secondary pathways typically activated by the receptor. Fortunately, both dLight and GRAB_DA_ have been shown to trigger no significant activation of the G-protein and β-arrestin signaling pathways associated with endogenous dopamine receptors.

Similar to iGluSnFR, both dLight and GRAB_DA_ transgenes can each be delivered via AAV vector for expression in the brain. Brain tissue expressing dLight1.2 examined as *ex vivo* brain slices demonstrate rapid kinetics, with a rise τ_1/2 _=** **9.5** **±** **1.1 ms followed by an ∼150 ms plateau peak and a decay of τ_1/2 _=** **90** **±** **11 ms. Brain tissue expressing GRAB_DA1m_ and GRAB_DA1h_ in *ex vivo* brain slices exhibit similar rapid kinetics, with GRAB_DA1m_ showing a rise τ_1/2 _=** **0.08 s and decay τ_1/2 _=** **3.12 s and GRAB_DA1h_ showing rise τ_1/2 _=** **0.11 s and decay τ_1/2 _=** **17.15 s. The GRAB_DA_ constructs are also noted to be significantly more photostable than iGluSnFR, enabling imaging over several hours.

Both dLight and GRAB_DA_ have demonstrated remarkable utility during *in vivo* measurements, allowing DA transients to be monitored in awake and behaving animals. dLight has shown robust performance in mice, reporting DA dynamics in response to pavlovian conditioning and reward. Similarly, GRAB_DA_ has demonstrated robust performance in mice in addition to other model species such as *Drosophila* and larval zebrafish. The viability of these platforms' performance *in vivo* has motivated the extension of this sensor platform to additional targets. A similar cGFP inserted receptor sensor has been developed and characterized for acetylcholine and demonstrated in both *Drosophila* and mice *in vivo,* while initial screens have identified promising sensor candidates for adrenaline, opioid, and serotonin sensing.

## III. SYNTHETIC PROBES

In contrast to protein-based neurotransmitter sensing methods, synthetic probes are characterized by their exclusive use of non-natural constructs to bind and signal neurotransmitters. Though each sensor maintains the basic structure of a molecular recognition element paired with a signal transducer, synthetic neurotransmitter sensing methods leverage a variety of molecular recognition and signal transduction elements taken from fields outside of traditional biology. As a result, these sensors employ a diverse range of strategies to achieve the desired goals of temporal resolution, sensitivity, specificity, and biocompatibility. To structure this collection of methods, we further organize synthetic neurotransmitter sensing methods into molecular-scale sensing methods and device-scale sensing methods. Sensors in both methods often incorporate similar molecular recognition or signal transducing moieties. However, the two are distinguished by their respective modes of operation. Device-scale methods typically organize their molecular recognition and signal transduction moieties on macroscopic structures such as chips or probes. Conversely, molecular-scale methods organize their molecular recognition and signal transduction moieties into molecular-scale units.

A noted challenge in designing synthetic neurotransmitter sensing methods is reconciling their nonbiological origins with their ultimately biological destination. Biocompatibility, lack of robustness in biological settings, unfavorable binding kinetics, and poor signal-to-noise ratio all present potential areas of sensor failure. Whereas most protein-based methods have been implemented broadly in *in vitro* cell cultures, in *ex vivo* brain tissue slices, and even within *in vivo* in animal models, many of the synthetic sensors we discuss in Sec. III A have not undergone similar levels of full implementation in the realistic biological milieu. Thus, synthetic neurotransmitter sensors that have been rigorously tested in biological milieus—both *in vitro* and *in vivo*—are underrepresented in the literature but remain of particular interest to both the neurotransmission imaging community and the materials science community.

### A. Molecular synthetic probes

Molecular synthetic probes are distinguished from device-based probes (discussed subsequently in Sec. III B) by their ability to be used in “free” forms that are not physically linked into a larger device, such as an electrode or a chip. Molecular probes may allow finer spatial resolution than their device-based counterparts through increased sensor density in, and accessibility to, smaller areas of neurobiological interest.

#### 1. Gold nanoparticles

Gold nanoparticles (AuNP), defined as particles with their smallest dimension ranging from 1 to 100 nm, have served as attractive imaging agents due to their tunable absorption and emission properties and access to a library of diverse surface chemistries.^20^ In particular, the ability of AuNPs to change in color in response to changes in plasmon resonance frequency makes them attractive scaffolds for colorimetric sensors. Colorimetric sensor responses can be observed with the naked eye, often bypassing the need for special instruments or microscopy methods. As a result, gold nanoparticles are attractive scaffolds for the development of sensors to be used in routine benchtop analyses.^21^ Many of the neurotransmitter sensor designs highlighted here that incorporate AuNP are largely directed toward sensing neurotransmitter concentrations in blood or serum.

In conjunction with the AuNP signal transduction moiety, each AuNP sensor we highlight utilizes an aptamer as a molecular recognition element. Aptamers are short polymers synthesized from peptides or nucleotide sequences and are capable of forming analyte-binding pockets from their secondary conformations. In comparison to analyte-binding proteins, aptamers are easier to synthesize with minimal batch-to-batch variation and are more chemically stable.^22^ As such, they frequently serve as functionalization moieties in synthetic probe development.

**Fig. 4.**
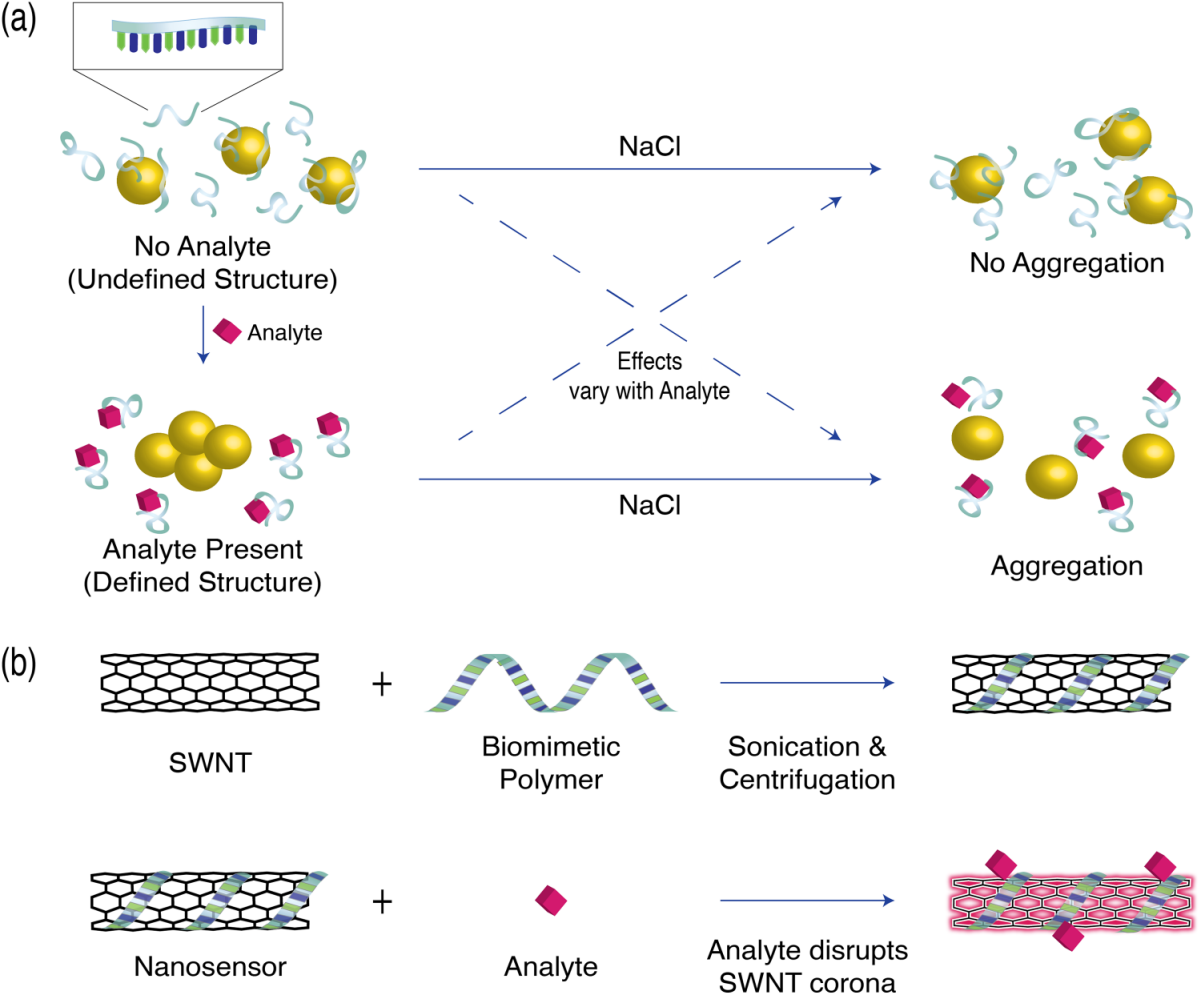
Schematic of molecular synthetic sensor mechanisms. (a) AuNP-DNA Aptamer systems adopt specific DNA aptamer formations in the presence of their analyte. Depending on the analyte, this can either shield the AuNP or expose AuNP to NaCl aggregation. (b) SWNT sensors are generated by wrapping SWNT in biomimetic polymer strands. Interaction between the sensor corona and analyte (pink diamond) results in fluorescence modulation.

The colorimetric AuNP-aptamer sensor designed by Zheng *et al.* incorporates a dopamine-binding DNA aptamer, with a K_d_ of 0.7 *μ*M, as a dopamine recognition element, and features unmodified gold nanoparticles as reporters of dopamine binding.^21^ In the absence of dopamine, dopamine-binding DNA aptamers are free to adopt a variety of conformations and adsorb onto the AuNP surface. This results in protection of the AuNP from aggregation in the presence of 0.25M NaCl.^21^ However, in the presence of dopamine, the dopamine-binding DNA aptamers are driven to form a defined structure and are no longer able to shield the AuNPs from aggregating and changing their plasmon resonance frequency [Fig. 4(a)]. This aggregation in turn generates a colorimetric response that scales with the concentration of dopamine^21^ and has demonstrated selectivity for dopamine over common competitors such as epinephrine and ascorbic acid. It is important to note that the sensor system has only been demonstrated in the proof of concept aqueous solutions of sensor and binding target, and the concept remains unexplored for tissue preparations or for *in vivo* implementation.

A similar AuNP-aptamer system has been developed by Chavez *et al.* to sense serotonin by substituting a serotonin-binding aptamer. However, the assay system observes an increase in aptamer shielding and decrease in AuNP aggregation upon addition of serotonin [Fig. 4(a)]. This strategy yields a linear sensor response in the physiologically relevant serotonin concentration range of 750 nM–2.5 *μ*M.^23^ Sensing tests done in serotonin-spiked fetal bovine serum (FBS) noted that the presence of proteins with the fetal bovine serum interfered with AuNP-aptamer serotonin response.^23^ Thus, samples were first filtered through amicon filters before AuNP-aptamer based sensing.

Further improvements on the sensitivity of the dopamine AuNP-aptamer sensor have been made by Cao *et al*. by leveraging surface plasmon resonance (SPR) to detect dopamine levels as low as 200 fM.^24^ This detection scheme is achieved by immobilizing a DNA probe complementary to the dopamine-binding aptamer on an SPR chip via a Michael addition reaction and chemically conjugating the dopamine-binding aptamer to the AuNP surface. In the presence of dopamine, the conjugated AuNP cannot bind the chip surface, and, thus, no signal is observed. Conversely, in the absence of dopamine, the AuNP aptamer can bind to its complementary strand on the chip surface and generate a large SPR signal.^24^ Similar to AuNP-aptamer sensor of Zheng *et al.*, SPR AuNP-aptamer sensor of Cao *et al.* shows specificity for dopamine binding, but has only be studied for dopamine concentration in phosphate buffered saline and remains to be explored for dopamine detection in more complex biological milieus.

Collectively, AuNP sensors have shown great promise as sensitive and easily interpretable neurotransmitter indicators. However, their limited testing in realistic biological environments remains a major challenge for their future development. Initial tests in FBS have indicated AuNP sensor attenuation when exposed to proteins, which are abundant in biological samples. Thus, at their current state, AuNP sensors remain best suited for *ex vivo* studies where samples can be pretreated to ensure robust sensor performance.

#### 2. Single wall carbon nanotubes

In contrast to the colorimetric response of gold nanoparticle-based sensors, single walled carbon nanotube (SWNT)-based sensors employ a signaling moiety that fluoresces in the near infrared (NIR) via exciton recombination along the one-dimensional nanoparticle. The NIR window provides considerable advantages in probing sensor response within tissues, both *in vivo* and *in vitro*.^25–27^ The NIR I region (650–950 nm) presents a local minimum in the tissue absorption spectrum that avoids the absorbance of hemoglobin and water; two major components within tissue that limit the depth at which tissue can be imaged at a high resolution.^28^ An additional transparency window, referred to as the NIR II region (1000–1350 nm), has also been identified experimentally and computationally to give even greater optical tissue penetration depth than the NIR I region.^28^ Given the reduced scattering and absorption for in-tissue imaging, much effort has been put toward developing fluorophores that fluoresce in the NIR regions. Thus, the NIR bandgap exhibited by single wall carbon nanotubes has been an attractive platform upon which to base sensors for probing biochemical events in tissue. An additional advantageous photonic property exhibited by SWNT-based sensors is their nonphotobleaching fluorescence, showing no attenuation in fluorescence even after 10 h of continuous excitation.^29^ This allows for samples to be observed repeatedly over extended time courses to probe long-term phenomena.

Leveraging the photonic properties of SWNTs for molecular recognition requires introducing a neurotransmitter recognition moiety that will modulate the SWNT fluorescence during target neurotransmitter binding. This is achieved by functionalizing SWNTs with biomimetic polymeric strands. Polymer functionalized SWNTs allow for regions of the polymeric strands to adsorb to the SWNT nanoparticle, creating a corona phase at the nanotube surface. The SWNT surface-adsorbed polymers interact with and selectively bind molecular targets, such as neurotransmitters, and generate changes in the nanotube corona that result in modulation of SWNT fluorescence [Fig. 4(b)].^30^

The most extensively studied SWNT-based sensor is a dopamine nanosensor constructed from adsorption of a single stranded DNA polymer composed of 15 guanine and tyrosine repeats (GT)_15_. *In vitro* solution phase studies have shown (GT)_15_-SWNT fluorescence signal increases up to 80% in the presence of 100 *μ*M dopamine, which translates to ΔF/F** **=** **0.3 at peak physiological dopamine concentrations that follow burst neuronal firing events (∼1 *μ*M). The (GT)_15_-SWNT nanosensor also exhibits a linear working range of 10 nM–10 *μ*M.^31^ More recently, another SWNT-DNA construct, (GT)_6_-SWNT, has been shown to yield fluorescence response (ΔF/F_0_) of up to 2400% in response to 100 *μ*M dopamine and shows selectivity toward a new neuromodulator target, norepinephrine, with ΔF/F_0_** **=** **3500% sensitivity and K_d_ values of 35 *μ*M for norepinephrine and 10 *μ*M for dopamine. Importantly, this nanosensor shows selectivity for dopamine and norepinephrine over other potentially competing and ubiquitous neurotransmitters, such as glutamate (Glu), Ach, and GABA. The (GT)_6_-SWNT nanosensor is shown to remain sensitive over a broad range of *p*H (4–9) and salt (1–200 mM NaCl) conditions, suggesting potential compatibility for *in vivo* use.^32,33^

The suitability of (GT)_15_-SWNTs to probe dopamine release has been examined *in vitro* with cultured PC12 cells by generating immobilized (GT)_15_-SWNT nanoarrays on (3-amino-propyl) triethoxysilane functionalized glass, followed by collagen deposition. The PC12 cells were cultured on the nanoarray, allowing cellular dopamine efflux profiles to be monitored by ∼20 000 sensors under the area of the cell body.^34^ This is a marked improvement over existing microarray methods for monitoring cellular analyte efflux, which provide 4–16 electrodes in the cell vicinity.^35,36^ This dramatic increase in spatial resolution is accompanied by (GT)_15_-SWNT reversible binding of dopamine with subsecond temporal resolution.^31,34^ This endows (GT)_15_-SWNT with a temporal resolution comparable to that of the current temporal standard of fast scan cyclic voltammetry (FSCV) (discussed in more detail below) in addition to the high spatial resolution.

A key element of neurotransmitter imaging involves monitoring both release and reuptake events. Whereas the above study enabled imaging of cellular dopamine release, it does not capture neurotransmitter reuptake mediated by endogenous transporter proteins. One recent study accomplished dopamine imaging in brain slices with highly sensitive (GT)_6_-SWNT constructs, which the authors named nIRCats (near-infrared catecholamine sensors). The authors implemented nIRCats in acute striatal brain slices to image dopamine release and reuptake and demonstrated micrometer scale spatial and subsecond temporal resolution in response to single pulse electrical stimulations.^37^ The high spatial resolution of nIRCats helped identify dopamine release hotspots based on nIRCat ΔF/F_0_ fluorescence and optimal binding kinetics allowed monitoring of dopamine reuptake process with temporal resolutions as fast as those observed with fast scan cyclic voltammetry. Furthermore, unlike dLight and GRAB_DA_ constructs which do not allow the use of antagonist and agonist drugs, nIRCats were demonstrated to retain their efficacy when used concurrently with a variety of receptor-targeting pharmacological agents, thereby affording imaging of dopamine neurochemistry in the presence of various drugs of relevance.^37^

#### 3. Fluorescent false neurotransmitters and FM dyes

**Fig. 5.**
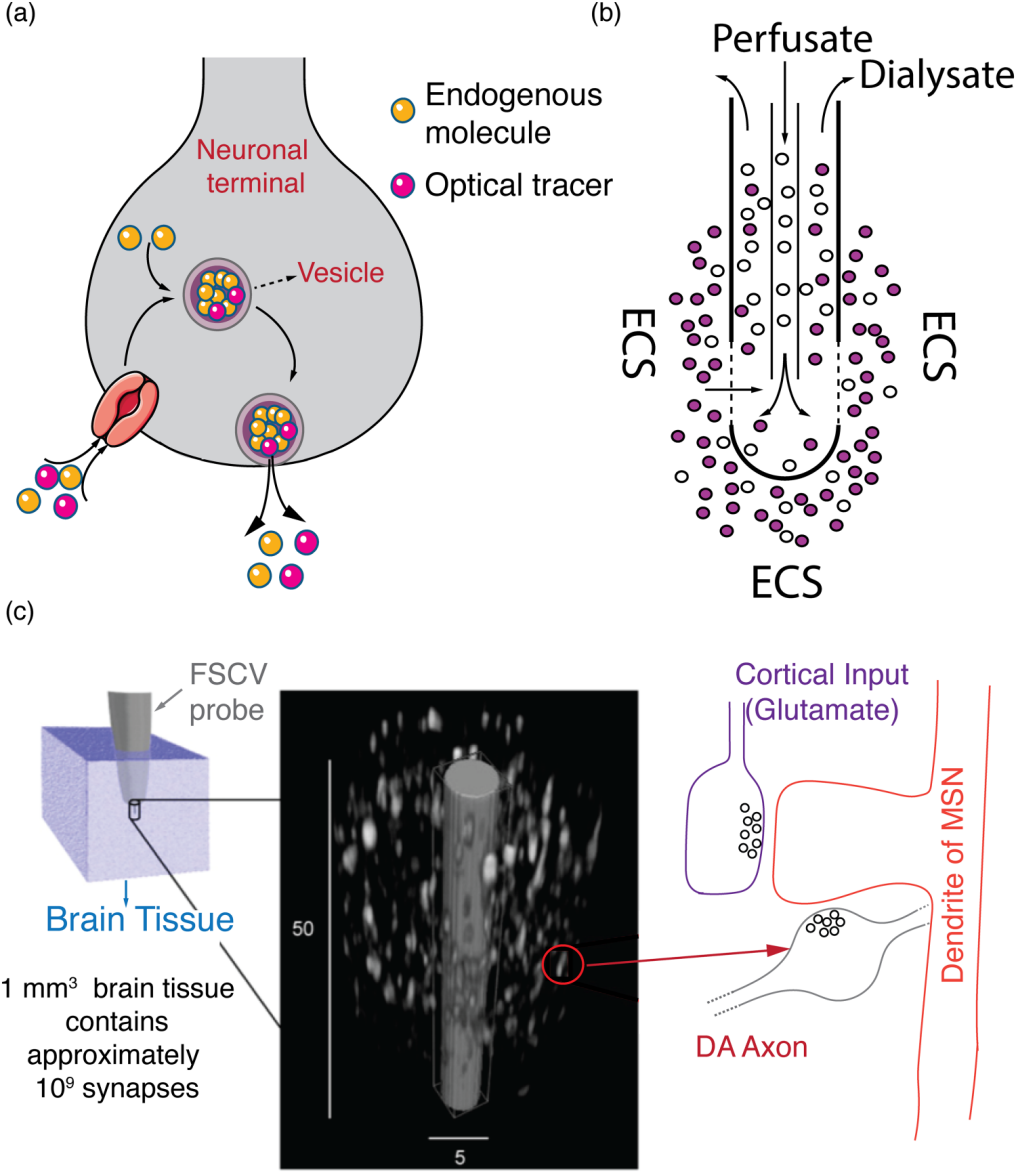
Fluorescent, analytical, and electrochemical methods for neurochemistry studies. (a) Schematic of FFN uptake and translocation into vesicles along with endogenous molecules. The FFN is fluorescent, enabling optical tracing of vesicular exocytosis with synaptic spatial resolution. The FFN (Optical Tracer) is internalized into the terminal along with endogenous dopamine molecules (Endogenous molecule). Inside the terminal, FFNs and dopamine molecules are cotranslocated into a vesicle, transported to the cell membrane and released into the ECS. (b) Schematic of a microdialysis probe. The blank perfusate typically contains no analyte of interest. Analytes diffuse across the semipermeable membrane and are recovered as dialysate for downstream analytical quantification. (c) Schematic of FSCV. The size of the FSCV probe (∼5 *μ*m diameter) is shown relative to the size of dopaminergic terminals in the striatum. Left panel: a 1 mm^3^ brain tissue is estimated to contain up to 10^9^ chemical synapses. Middle panel: a closeup of the tip of an FSCV probe (gray) is shown relative to the size of dopaminergic terminals (white) from which it is sampling. Only dopamine terminals are shown for clarity. Right panel: a representative dopaminergic terminal is shown on the right forming a synapse onto the dendritic spine of a medium spiny neuron. As is typical in the striatum, where FSCV probes are widely used, a glutamatergic terminal is also shown forming a synapse onto the same dendritic spine. (c) is reprinted with permission from D. Sames, M. Dunn, R. J. Karpowicz, and D. Sulzer, ACS Chem. Neurosci. **4**, 648 (2013). Copyright 2013, American Chemical Society.

Fluorescent false neurotransmitters (FFNs) and FM dyes (named after their inventor Fei Mao) are synthetic fluorescent probes that have been utilized to visualize neurotransmitter release dynamics. When an action potential arrives at a neuron's synaptic terminal, it induces the release of neurotransmitters that travel across the synaptic cleft and activate receptors on the postsynaptic neuron. This converts the action potential's original electrical signal into a chemical signal. To accomplish this complex event, neurons package neurotransmitters into nanometer-scale vesicles, which get released into the synaptic cleft following action potential mediated membrane fusion [Fig. 5(a)]. This chemical communication is rapidly terminated by membrane proteins (known as transporters) that efficiently clear the released signaling molecules from the synaptic cleft and prepare the synapse for subsequent action potentials (and neurotransmitter releases). Once inside the cytoplasm, recycled and newly synthesized neurotransmitter molecules are translocated into vesicles by vesicular transporter proteins, where they undergo subsequent biochemical processing for future release. To visualize the neurochemical release portion of this cycle, FFNs were developed to track the exocytosis of neuromotransmitters. FFNs are exogenous fluorescent molecules that contain moieties recognized by the neuron's transport proteins and are taken up and packed into vesicles along with their endogenous counterparts [Fig. 5(a)]. As such, FFNs enable visualization of the release of neurotransmitters with single release-site (i.e., synaptic) resolution and provide fundamental insight into synaptic release probability, a parameter that is important for characterizing synaptic plasticity.

While the principle behind FFNs is widely applicable, to date, only FFNs for the neuromodulator dopamine have been well characterized. The vesicular transporter for dopamine, VMAT2, has a broad affinity for molecules with ethylamine side chains and is the endogenous transporter for norepinephrine and serotonin, in addition to dopamine. VMAT2 is known to transport other exogenous substrates with ethylamine side chains, such as amphetamine and tyramine. Gubernator *et al.*^38^ take advantage of this VMAT2 promiscuity to design an optical tracer for dopamine, FFN511, in which an ethylamine side chain is covalently linked to a fluorescent coumarin core. FFN511 is efficiently taken up by dopaminergic neurons and packaged into vesicles along with endogenous dopamine molecules, as demonstrated in cell cultures as well as brain tissue slices. Upon potassium and electrically induced stimulation of FFN-labeled tissue, FFN511 and other similar constructs (FFN200, FFN202, FFN102) have enabled visualization of dopamine vesicular exocytosis with synapse-scale spatial resolution.^38–41^ Using a similar approach, an optical tracer for serotonin (FFN 246) has shown modest success as well.^42^

In contrast to FFNs, FM dyes do not have a specific affinity for transporter proteins. Rather, they possess highly lipophilic moieties that efficiently partition into plasma membranes and other hydrophobic domains in the cytoplasm. Their fluorescent moiety is sensitive to the polarity of the solvent such that when they are trapped in hydrophobic domains; their fluorescence is amplified by more than two orders of magnitude. These two properties make them efficient at labeling plasma membranes and other endosomal structures, including vesicles into which neurotransmitters are packaged before release. The styryl based FM1-43 dye has been particularly well suited for observing vesicle formation and membrane turnover during endocytosis, for monitoring vesicle trafficking inside synaptic boutons, and for visualizing exocytosis of neurotransmitter-containing vesicles during action potentials.^43–45^ FM dyes have achieved widespread use owing to their low toxicity, their simplicity of use, and the generic nature of their application. They are commercially available, and their fluorescence spectrum spans an optical range whose breadth affords dual-color imaging. To integrate FM dyes into vesicles or vesicular membranes, the tissue must undergo electrical stimulation to derive vesicle exocytosis, with the consequent compensatory endocytosis producing labeled endosomal structures and vesicles. This is a relatively invasive dye loading technique in contrast to other optical sensors and probes where molecules are genetically expressed in target cells or are loaded by passive incubation methods.

Both FFNs and FM dyes exhibit two noteworthy shortcomings: lack of direct neurotransmitter sensing and minimal use *in vivo*. Due to their design, FFN and FM fluorescence does not directly report on local neuroanalyte dynamics. Rather, FFN and FM dyes function by integrating into the cellular machinery that is involved in neurotransmitter packing and release and serve as a proxy for the physiological process and its underlying neurochemistry. As such, they provide little information about the extracellular dynamics of released neurotransmitter molecules, the spatial extent of their diffusion, dynamics of neurotransmitters reuptake from the brain ECS, or how neurochemistry is affected by drugs or disease. Furthermore, FFNs and FM dyes have been used almost universally in reduced preparations such as acute brain slices or neuronal cell cultures and are yet to be demonstrated for routine use *in vivo*.

### B. Device-based probes

In addition to fluorescent reporting methods discussed in Secs. III A 1–III A 3, other tools for measuring the concentration of chemical analytes in the brain exist. The device-based methods we highlight below do not provide an optical readout and offer limited spatial information. However, device-based methods can provide a temporal response that can outperform the temporal response of certain optical methods discussed in Secs. III A 1–III A 3. Here, we highlight two technologies that predate the use of fluorescent chemical probes for studying neurochemistry. First, we discuss microdialysis, one of the earliest methods developed for identifying and quantifying analytes in the brain's extracellular space. We then discuss electrochemical methods that have been extensively used for studying redox-active molecules in the brain. For both techniques, we highlight the principles of operation and contextualize their application for neuroscience research by drawing parallels in temporal and spatial resolution, specificity, and multiplexing capability. This review of the workhorse device-based methods within neuroscience is then followed by the examination of modern field effect transistor (FET) devices currently being developed.

#### 1. Microdialysis

One of the earliest methods employed for characterizing brain neurochemistry is microdialysis, a technique in which samples from the interstitial space of brain tissue are recovered by dialysis and characterized using established analytical approaches such as liquid chromatography, capillary electrophoresis, mass spectrometry, and electrochemistry.^46–48^ Microdialysis involves the insertion of a dialysis probe with a tubular semipermeable membrane into relevant brain region of interest in anesthetized or awake animals. Once inserted, the probe is perfused with a solution (blank perfusate) whose ionic balance resembles that of the extracellular fluid. The term “blank perfusate” refers to the fact that the perfusate is devoid of the small molecule analytes that are of interest to the experimenter, yet is iso-osmotic with cerebrospinal fluid with monovalent and divalent ions that are necessary for normal brain function (Na^+^, K^+^, Ca^2+^). Maintaining proper ionic balance ensures that the homeostatic balance of neural tissue surrounding the probe will not be adversely impacted by ionic concentration gradients across the dialysis membrane. Meanwhile, small molecule analytes, such as neurotransmitters, hormones, and metabolites, can freely diffuse from the extracellular space into the perfusate along their concentration gradient. The continuously flowing perfusate is then collected via outlet tubing for further analytical quantification [Fig. 5(b)].

Microdialysis' greatest advantage is its ability to provide highly selective and reliable identification of a wide range of molecules, including neurotransmitters, neuropeptides, and hormones.^46^ Microdialysis is easily expanded to a wide range of analytes without concern for ligand binding affinities and inherently multiplexes to sample for the simultaneous analysis of many analytes. To this end, an extensive body of research has demonstrated the versatility of microdialysis in the types of analytes that can be analyzed, as well as their location of measurement. These include neurotransmitters such as dopamine, glutamate, GABA, and histamine; neuropeptides such as oxytocin, Substance P and neurokinin; hormones; and other signaling molecules, such as somatostatin, prolactin, and cyclic AMP.^49–54^

Microdialysis has been repeatedly demonstrated as a powerful tool for *in vivo* applications within many regions of the brain.^46^ Once implanted, the dialysis probe can be repeatedly used for chronic analysis for behavior studies. In retrodialysis, a particular method of microdialysis, small molecule pharmacological agents can be delivered into the extracellular space by passive diffusion across the dialysis membrane from the perfusate. Modern analytical techniques have been developed in which real-time measurements of the neuroanalytes subject to the pharmacological intervention can be made available to the experimenter.^46,48^

Despite its versatility, microdialysis also exhibits several drawbacks, the most important of which is a poor temporal resolution.^46,48^ Low perfusion flow rates are required to allow analytically quantifiable diffusion of analytes into the perfusate. As such, in most microdialysis setups, samples are recovered approximately once every 10 min. Fastest reported time resolutions using the most modern setups still exceed 1 min.^55,56^ Consequently, microdialysis cannot capture fast dynamic events that are a hallmark of most brain neurochemistry. As such, the use of microdialysis is most suited to studying changes in basal levels of analytes caused by processes that occur on the scale of several minutes, hours, or days.

The efficiency of analyte extraction also serves to be a major challenge. Typically, fast perfusate flow rates are advantageous from a temporal resolution perspective but lead to low analyte recovery resulting in significant differences in analyte concentration in the dialysate and in the extracellular space and may fall below detection limit of the analytical equipment. The ratio of analytes in the dialysate to the actual tissue concentration, known as extraction efficiency, is therefore sensitive to and inversely related to perfusate flow rate. In principle, it is possible to slow down the perfusion speed to achieve equilibrium between the dialysate and the neurochemical contents of the ECS. This, however, comes at the cost of reduced temporal resolution that may run afoul of the experimenter's objectives and add significant wait times between sample data points. Thus, results from microdialysis measurements, especially for neurotransmitter concentration measurements in the brain, could differ substantially from the true analyte concentrations in tissue.^48^

The spatial resolution of microdialysis is primarily limited by the size of the probe. Most microdialysis probes have lengths on the order of millimeters and widths on the scale of several hundreds of micrometers, affording only sampling of analytes from volumes that span hundreds of micrometers from the probe. Dialysis probes also require surgical implantation, which can result in mechanical damage to the tissue surrounding the probe. The limited spatial information provided by microdialysis and other device-based techniques is a primary disadvantage compared to smaller sensors, such as molecular fluorescent probes, which provide more detailed readouts across space. Finally, recovering samples using dialysis from the extracellular space could lead to analyte depletion from the tissue surrounding the probe. Analyte depletion originates because the perfusate is devoid of neurochemical analytes, giving rise to large concentration gradients across the dialysis membrane. It is widely understood that this phenomenon can lead to altered neurochemical dynamics in the tissue surrounding the probe. Zero-net-flux microdialysis, in which the perfusate is prepared with a predetermined concentration of the analyte to minimize or eliminate analyte concentration gradients, is employed to minimize impacts of analyte depletion in tissue surrounding the probe.^46,48^ In sum, the primary strength of microdialysis is in the high chemical specificity of the technique, with disadvantages including low spatial resolution, analyte depletion, low temporal resolution, and experimental variations that are functions of probe size and placement, experimental design and data analysis and interpretation.

#### 2. Electrochemical methods

Voltammetry and amperometry are electrochemical methods that are widely used for quantifying the neurochemistry of specific redox-active molecules, such as dopamine, norepinephrine, and serotonin, as well as their metabolites.^57^ These methods provide a tremendous improvement in temporal resolution, and in contrast to microdialysis, can measure millisecond-scale transients in analyte concentration. The principle behind these methods borrows from an analytical technique in chemistry, in which appropriately applied voltage at the tip of a microelectrode oxidizes certain analytes. The current generated by the redox chemistry can be measured and quantitatively related to concentration, serving as a readout for local analyte concentration. Typically, the applied voltage is varied as a function of time within a specific potential window, and the corresponding current-voltage plot, known as voltammogram, serves as a readout for both analyte concentration and chemical identity. The rapid sweep of potential at the working electrode enables identification of transients in *specific* brain analyte concentrations with high temporal resolution. When used as such, the technique is known as FSCV.^58^

FSCV has been used extensively to study a group of neurotransmitters known as catecholamines, with extensive applications for the study of dopaminergic systems in particular.^59^ Dopamine projections into the dorsal striatum and nucleus accumbens form large subcortical structures that exhibit a uniform distribution of dopamine terminals (dopamine release sites). The uniformity of dopaminergic terminal distribution, dense dopamine innervation, and the specificity with which dopamine can be identified have all contributed to the wide application of FSCV for studying dopamine neuromodulation. The fast temporal resolution of FSCV, in which cyclic waveforms are applied as fast as ten times per second, allows measurement of transient spikes and clearances in dopamine concentration, affording accurate recapitulation of the underlying dopamine chemodynamics. However, owing to the relatively large size of FSCV electrodes, regions of the brain in which dopamine has sparse terminal distribution, such as the neocortex, are not as amenable to FSCV measurement.

Serotonin is a similarly electroactive molecule that is amenable to interrogation with FSCV.^60^ Like dopamine, serotonin signals mostly through GPCR receptors are widely expressed throughout the brain and have a powerful effect on the etiology and treatment of depressive disorders, making it a molecule of intense interest in neuroscience research.^61^ Although serotonergic cell bodies from the dorsal raphe nucleus project widely and diffusely to many regions of the brain, the application of FSCV to serotonergic systems is limited to regions that exhibit robust and dense innervation, such as the substantia nigra pars reticulata, and certain hypothalamic and thalamic regions.^61^

Many neurotransmitters of critical interest in neuroscience, such as glutamate and acetylcholine, are not inherently electroactive and hence are not amenable to detection by electrochemical methods. This disadvantage is often circumvented by the synthesis of enzyme-coated microelectrodes. The approach here couples the electrochemical principles described previously with oxidase enzyme-coated microelectrodes, in which the enzyme acts on the substrate of interest (in this case neurotransmitters) and selectively generates reactive oxygen species (often hydrogen peroxide) that can in turn be detected electrochemically. The enzyme coating affords selective electrochemical response from otherwise nonelectroactive molecules. Glutamate oxidase coated microelectrodes have been demonstrated for measuring glutamate release *in vivo*.^62^ In the same vein, coimmobilized acetylcholine esterase and choline oxidase have been demonstrated for successful detection of the neurotransmitter acetylcholine.^63,64^ However, enzyme-coated amperometric methods require a substantial degree of finesse in electrode manufacturing and enzyme immobilization, where enzyme denaturation or fouling is also points of concern. Furthermore, enzyme-functionalized FSCV methods exhibit markedly slower temporal resolutions of ∼10 s compared to FSCV applied to inherently electroactive molecules such as dopamine, which have temporal resolutions on the order of ∼0.1 s.

In addition to temporal resolution, FSCV provides much improved spatial resolution compared to microdialysis. Carbon fiber electrodes can be constructed at the scale of single-digit micrometer width scales, resulting in improved spatial resolutions of more than an order of magnitude compared to microdialysis. The relatively small size of the working electrode also means that FSCV implementation, although still more invasive than injectable fluorescent neurochemical probes, is improved compared to the tissue damage that results from microdialysis probes. Chronically implanted microelectrodes have been successfully employed for long-term *in vivo* studies. However, while FSCV achieves significant improvement in spatial resolution compared to microdialysis, its spatial resolution remains limited to the immediate vicinity of the probe, unlike molecular fluorescent probes highlighted in Secs. II and III A. The smallest FSCV probes are still over an order of magnitude larger than the synaptic milieu into which neurotransmitter molecules are released. As such, FSCV is suitable for measuring ensemble averaged activity arising from stimulation of hundreds of dopaminergic neuronal terminals (sites of neurotransmitter release) at a time, and cannot reliably detect heterogeneous single synapse activities. This obligate averaging significantly restricts the application of FSCV to regions of the brain that exhibit dense and robust innervation of the target molecule. As such, despite well-known projections of dopamine into the prefrontal cortex, FSCV measurements of dopamine are mostly employed to study projections in the striatum. Correspondingly, FSCV measurements of serotonin are limited to areas with dense innervation despite serotonin's wide and diffuse distribution to many parts of the brain.

#### 3. Field effect transistor-based probes

FETs are devices that utilize changes in the electric field to drive device performance. As a result, there are a number of device-based sensors that have utilized FETs to sense changes in electric fields induced by neurotransmitter binding. In comparison to optical or electrochemical signal transducers, FETs often have low analyte concentration detection limits.^65^ However, FETs readily interface with other biomedical devices and can leverage existing microfabrication techniques to quickly and inexpensively generate new sensors.

FETs can be interfaced directly with biological binding domains by isolating the lipid membranes of cells expressing a neurotransmitter's endogenous receptor. This strategy is leveraged by Kim *et al.* to develop an acetylcholine FET sensor by engineering *E. coli* to express the M1 muscarinic acetylcholine receptor (M1-mAchR). The lipid membranes of the *E. coli* are then collected and distributed over a FET made by adsorbing single wall carbon nanotubes to a silicon oxide substrate patterned with gold-palladium source and drain electrodes.^66^ In *p*H 7.4 buffer solution, acetylcholine has a positive charge of +1. Thus, it is postulated that upon binding to the M1-mAchR above the source and drain electrodes, the receptor-neurotransmitter complex presents a more positive gate bias on the FET, decreases the source-drain current, and generates a signal from the acetylcholine binding.^66^ The resulting acetylcholine FET sensor has a wide dynamic range of 100 pM–10 *μ*M and exhibits a dissociation constant K_d_ of 16 nM.^66^ In addition, the sensor shows marked sensitivity for acetylcholine over competing neurotransmitters such as GABA, glutamate, choline, and dopamine, though it has not been tested in more complex biological milieu.

FET sensors have also been developed using DNA aptamers rather than protein receptors as recognition moieties, as is the case for a dopamine FET sensor developed by Li *et al.*^67^ In comparison to protein receptors, aptamers are smaller and thus able to bring bound dopamine molecules closer to the FET surface to generate stronger changes in the electric field and thus stronger signals. Furthermore, aptamers can often demonstrate greater thermal stability than their protein counterparts and can self-refold into their correct conformations.^67^ Li *et al.* also opt for a multiple-parallel-connected silicon nanowire FET with interdigitated source and drain electrodes over the carbon nanotube design used by Kim *et al*. and directly immobilize the neurotransmitter binding aptamer to the FET's silicon nanowire surface.^67^ The final FET dopamine sensor demonstrates a similarly wide linear working range of 10 pM–100 nM, a dissociation constant K_d_ for dopamine of 120 pM, and a 50- and 10-fold preferred affinity for dopamine over epinephrine and norepinephrine, respectively.^67^ These specifications have allowed real-time observations of fluctuating dopamine efflux from PC12 cells in hypoxic conditions.^67^

Banerjee *et al.* have further developed silicon nanowire FET designs of Li *et al*. Most notably, this work demonstrates the development of an FET-aptamer sensor for Neuropeptide Y (K_d_** **=** **295 nM) and the use of the FET dopamine and FET Neuropeptide Y sensors together to monitor temporal release profiles of dopamine and Neuropeptide Y from PC12 cells stimulated with histamine.^68^ This ability to simultaneously monitor dopamine and Neuropeptide Y together is of particular note, as neuropeptides are typically monitored through methods with poor temporal resolution, such as western blots, and microdialysis-coupled analytical methods such as mass spectrometry.

Although microfabrication processes for silicon nanowire and carbon nanotube FETs are relatively established, the expensive machinery and clean fabrication environment required can also pose challenges to broad-scale implementation. In response to this, an alternative fabrication method was developed utilizing simple sol-gel chemistry and spin coating to generate In_2_O_3_ films of ∼4 nm in thickness. Electrode patterns can then be defined using self-assembled monolayers of alkanethiols on gold as soft masks for chemical lift-off lithography.^65^ FETs created through this process were then made into dopamine sensing FETs using the same DNA aptamers used by Li *et al.* and shown to recreate the 10 pM–100 nM working range previously identified.^65,67^

## IV. SUMMARY AND CONCLUSIONS

The electrical activity of neurons gives rise to chemical signaling, and the chemical signals in turn derive or tune the electrophysiology of the neurons that release the chemicals, creating a complex feedback mechanism in which the neurochemical state determines a wide range of attributes in brain function and dysfunction. Recording the electrical activity of one neuron or a small group of neurons at a time using electrophysiology tools, such as patch clamp, fails to provide the appropriate scale needed to decipher how widely distributed neuronal networks contribute to the formation of emergent phenomena such as memory, learning, and behavior. To gain a holistic understanding of brain neurochemical function, scalable tools that can measure neurochemistry at spatial and temporal scales commensurate to brain function are needed. Fluorescent protein-based probes have a high spatial and temporal resolution, and when coupled to genetic engineering tools, are able to target specific neuronal populations with great precision. Of note, most of the fluorescent probes that have been successfully demonstrated *in vivo* are protein-based. In contrast, a wide range of synthetic fluorescent probes show modest success *in vitro* in cell cultures and other reduced preparations; however, few have demonstrated *in vivo* applicability. We attribute this to the challenge of ensuring that synthetic probes remain biocompatible and retain their function when exposed to the complex biological milieu found *in vivo*. For synthetic approaches to be successful, a wide range of design parameters have to be satisfied, including high signal-to-noise ratio for video-rate fluorescence imaging, optimal binding kinetics, photostability, and low cytotoxicity. Despite their relatively low spatial resolution and lack of cellular specificity, device-based techniques such as FSCV and microdialysis have contributed a great deal to the literature of neurochemistry. New tools for querying neurochemistry will likely engender the next round of insights into our understanding of brain function, thereby offering tremendous opportunities for synthetic chemists, nanotechnologists, and protein engineers to be at the forefront of this endeavor.
